# Profiles and Perspectives in Particle Therapy: James M. Slater, MD, FACR: A conversation with Dr. Slater's science writer, William Preston, EdD.

**DOI:** 10.14338/IJPT-18-00049.1

**Published:** 2019-03-21

## Early Influences

According to Dr. James Slater's colleague, William Preston, Dr. Slater often remarked that he was a physics student before turning to medicine. “Physics teaches you how to think,” he liked to say, and his professional journey can be described as a largely self-directed process of learning, investigating potential means of delivering heavy-charged-particle treatments in hospital settings, and arriving at his own conclusions.

Dr. Slater loved solving problems; his enthusiasm for physics arose from the realization that physics and mathematics were excellent problem-solving tools. He also spoke admiringly of several people who influenced his life, and specifically his ability to solve problems. One was his third-grade teacher and two were professors of physics at the University of Utah.

Well aware of the work of Ernest O. Lawrence, Cornelius Tobias, and Robert Wilson from his early studies at the university, his initial professional aim, following a brief consideration of engineering (his father and uncles were engineers), was to become a physicist. He then began to learn about research with charged and neutral particles. He was strongly influenced by Dr. Wilson's seminal 1946 paper on the medical uses of protons, and he followed with interest Wilson's development of Fermilab and its medical facility, which was initially intended to be a proton facility but became a neutron facility at the urging of Chicago-area physicians. Dr. Slater similarly admired Dr. Cornelius Tobias for his work on protons at Berkeley in the mid-1950s, and later with helium and other heavier ions.

**Figure:**
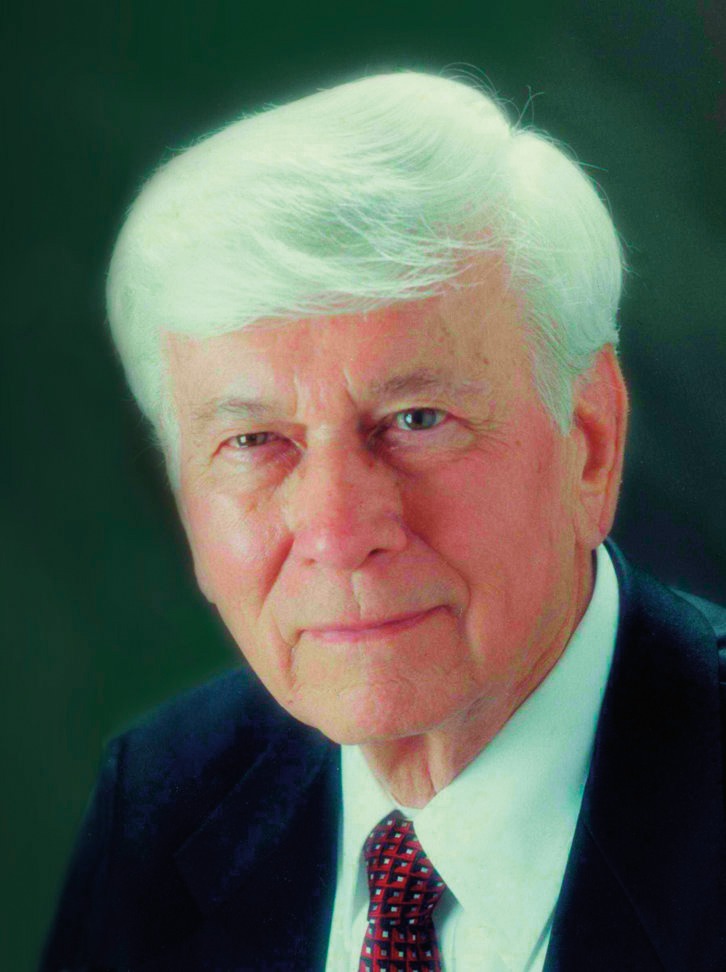


## Career Turning Points

It was by getting to know the physicians and pediatricians at Loma Linda, whom Dr. Slater met as a young parent, that he eventually came to understand that medical diagnosis is another form of problem solving. After deciding to pursue a career in medicine, he served a fellowship under Dr. Gilbert Fletcher at M.D. Anderson Cancer Center in the late 1960s. He admired Fletcher because he was an innovator, willing to think problems anew and try new solutions.

It is unclear when Dr. Slater decided to focus on radiation oncology, but he frequently suggested that radiology and radiation therapy were the fields in which he could best apply his knowledge of physics.

A major turning point occurred early during his residency training in radiotherapy in the mid-1960s: He was dismayed by the distress so many patients experienced during their X-ray treatments, in addition to, or sometimes apart from, their cancers. From his knowledge of physics and the behavior of X-rays, he knew that irradiation of too much normal tissue could lead to side effects that sometimes caused more suffering than the cancer. He became determined to develop new therapy planning techniques to limit normal-tissue exposure, mainly by making treatment set-ups more accurate and reproducible, given the limits imposed by technology at the time.

His early attempts to improve the precision of treatment delivery confirmed for him that two essential conditions must be met to reduce side effects: There must be a more accurate and precise way to image and identify the tumor and its extent of spread; and there must be a mode of delivery that could fully exploit more-precise treatment planning once it became available.

He began work on the former problem in the late 1960s as a resident at Loma Linda University. He consulted with a physicist at the university, Dr. Ivan Neilsen, about ways of incorporating patient imaging information directly into a treatment planning system. X-rays, as analog data, could not be used, but ultrasound data, which are digital, could. Accordingly, Drs. Slater and Neilsen, alongside two engineering colleagues, began developing an ultrasound-based therapy planning system (CT was not then available).

In 1970, Dr. Slater was recruited back to his medical alma mater, Loma Linda University, to develop a dedicated section of radiation oncology in the department of radiology. By that time, he desired a proton, pion, or other heavy-charged-particle facility for the new section and went so far as to support a study of its feasibility. That study revealed that the time was not yet right for such a facility in a hospital setting, owing mainly to the lack of adequate tumor imaging, treatment planning systems, and computer capability to support a heavy-charged-particle accelerator and treatment system. Dr. Slater then concentrated on completing the ultrasound-based planning system that he had been working on while a resident. That system was put into operation in 1971. It was refined thereafter, most notably when CT scanning became available and it could be linked with the planning system. The CT-based planning system garnered worldwide acclaim; one noted British radiation oncologist termed it the “missing link” in radiotherapy.

These efforts in therapy planning garnered honors for Dr. Slater and his colleagues: among them the first-place award for their exhibits “Computerized Radiotherapy Planning System” at the Scientific Exhibition of the Third Congress of the European Association of Radiology, Edinburgh, Scotland, June 1975, and “Computerized Tomography Scanning in Radiation Planning” at the annual meeting of the American Society of Therapeutic Radiologists, Los Angeles, California, November 1978.

Throughout the 1970s and early 1980s, Dr. Slater undertook a long-term plan of studying various particles and their suitability for routine radiation therapy. He treated patients with pions at Los Alamos National Laboratory and worked closely with the helium-ion and heavy-charged-particle groups at Berkeley. He consulted regularly with investigators at Massachusetts General Hospital and Harvard Cyclotron Laboratory as they pursued studies of proton radiation therapy. By the mid-1980s, he was convinced that the proton was the preferred particle for routine radiation oncology. By that time, some of the restraints that had deterred a hospital-based therapy center in 1970 had also been overcome; Dr. Slater proceeded to advocate for and develop the world's first hospital-based proton treatment center at Loma Linda.

## Greatest Interests and the Search for the ‘Ideal Beam'

Until recently, Dr. Slater was involved in several projects investigating new uses for proton therapy and optimizing its current uses in cancer care. His greatest interest was in expanding, and better understanding, how protons could be employed. As examples, he was involved in research using narrow proton beams to manage the intractable pain syndromes often encountered by victims of battlefield injuries; and he was also long involved in research on radiation sensitizers and radioprotectors, both aimed at increasing the effectiveness of protons either by sensitizing cancer cells, in the one instance, and protecting normal cells in the other.

Because of his pioneering role in developing hospital-based proton therapy, Dr. Slater could have taken on the “grey eminence” role, an idea he likely would have considered laughable. For him, the only proper role for a physician and scientist was to be in constant search of a better way to help patients. This had always been his underlying motivation.

Dr. Slater considered carefully every step he took toward development of the proton center at Loma Linda. He spent years studying and testing his ideas, then acting on his findings.

When he was overseeing the development of the center, critics suggested he instead develop a heavy-ion facility, arguing that he could achieve a greater biologic effect while still exploiting the Bragg peak. One of these critics was Dr. Cornelius Tobias. But Dr. Slater, as much as he respected Dr. Tobias, thought it better to use low-LET protons for routine radiation therapy. The *sine qua non* of treating patients with ionizing radiation is to spare as much normal tissue as possible. Heavier ions, which have a greater biologic effect in tumor tissue, also have the same effect in normal tissue. Despite the Bragg peak, normal tissues cannot be avoided entirely; accordingly, normal tissues exposed to a beam of heavy ions would suffer irreparable damage. Dr. Slater believed that, as with X-rays, proton beams permit tissues that are unavoidably irradiated to have a chance for self-repair.

Dr. Slater often spoke of the “ideal beam,” which would be one that delivers an optimum dose to tumor cells while avoiding every normal cell. Such an external beam does not exist and likely never will; failing that, then, he maintained that a low-LET beam that exploits the Bragg peak is the best available approximation.

## A Witness to Change

It pleased Dr. Slater to see the “experimental” label largely disappear from discussions of proton therapy.

In 1988, shortly after the groundbreaking ceremony had been held for today's James M. Slater, MD, Proton Treatment and Research Center, it was common to hear proton therapy described as experimental. Dr. Slater frequently said that such a description was untrue even then; the experimental work had been done in physics laboratories long before. Nonetheless, that perception persisted until Dr. Slater and his colleagues demonstrated that proton therapy could exist as a routine part of a hospital radiotherapy department.

Another significant change that Dr. Slater often mentioned is the growth of proton therapy worldwide, as shown by the number of centers in operation, under construction, and in planning stages (see https://www.ptcog.ch/). When the Loma Linda center opened in 1990, it was the first hospital-based proton therapy facility in the world, and it was the only one in the United States until the Francis H. Burr Center opened a decade later at Massachusetts General Hospital. Today, however, there are scores of such facilities, which Dr. Slater saw as a great resource for patients and for research.

## The Future of Particle Therapy and Patient Care

Dr. Slater long viewed research as fundamental to improving patient care. Although the proton beam offers inherent benefits to patients owing to our ability to control its dose deposition, he never regarded that capability as an end in itself.

Dr. Slater actively explored ways to optimize proton therapy and extend its use beyond oncology. He believed that the increase in proton centers offers many opportunities for collaborative trials and information sharing, allowing the benefits of particle therapy to be shown through large-scale data registries and multi-institutional studies. It is largely through the weight of evidence from large, collaborative trials that the value of proton therapy will be realized and the benefits made obvious to the general medical community.

While Dr. Slater never publicly discussed the role of organizations like the National Association for Proton Therapy, it is likely he saw such organizations as facilitators of collaborative research to improve particle therapy for patients.

## Advice for the Next Generation

As a scientist at heart, Dr. Slater believed in forever asking questions, forming hypotheses, and testing them. According to Preston, he would urge residents to be scientists, or to at least employ a scientist's mentality. Backed by their knowledge, scientists should use their imaginations when determining the best ways to implement therapeutic tools.

Dr. Slater relied on his imagination when contemplating matters such as cancer spread patterns and the behavior of accelerated particles in tissue. Contemplation led him to exploratory ideas and research problems from which he derived testable hypotheses about better ways to use proton therapy.

Always look for better ways, he would say. Never be satisfied that current methods are as good as they can be for patients.

